# Occurrence and Exposure Assessment of Aflatoxins and Deoxynivalenol in Cereal-Based Baby Foods for Infants

**DOI:** 10.3390/toxins11030150

**Published:** 2019-03-05

**Authors:** Marta Herrera, Noemi Bervis, Juan José Carramiñana, Teresa Juan, Antonio Herrera, Agustín Ariño, Susana Lorán

**Affiliations:** 1 Instituto Agroalimentario de Aragón—IA2 (Universidad de Zaragoza-CITA), Facultad de Veterinaria, 50013 Zaragoza, Spain; herremar@unizar.es (M.H.); nbervis@unizar.es (N.B.); carramin@unizar.es (J.J.C.); aherrera@unizar.es (A.H.); sloran@unizar.es (S.L.); 2 Centro de Investigación y Tecnología Agroalimentaria de Aragón (CITA), 50059 Zaragoza, Spain; tjuan@cita-aragon.es

**Keywords:** mycotoxins, aflatoxins, deoxynivalenol, infant cereals, baby food, maximum levels

## Abstract

Aflatoxins are carcinogenic to humans and deoxynivalenol causes digestive disorders, and both mycotoxins occur frequently in cereal-based foods. The purpose of this study was to investigate the occurrence and levels of aflatoxins (B1, B2, G1 and G2) and deoxynivalenol (DON) in cereal-based baby foods as well as to calculate the estimated daily intakes (EDI) in different stages of infancy. Sixty samples of infant cereals (wheat-, corn-, rice-, oat-, and mixed grain-based) were collected during a 2-year period and analyzed by validated methods. Aflatoxins were detected in 12 samples (20%), six of which exceeded the EU maximum level for aflatoxin B1 set at 0.10 µg/kg. Deoxynivalenol appeared in 20% of baby food samples, with one sample exceeding the EU maximum level established at 200 µg/kg. There were no significant differences between gluten-free products for babies aged 4–6 months and multi-cereal products for infants aged 7–12 months, nor between whole-grain-based and refined ingredients. However, baby food products of organic origin showed significantly higher levels of deoxynivalenol than conventional ones (*p* < 0.05). It is proposed for the health protection of infants and young children, a vulnerable group, to establish the lowest maximum level for the sum of aflatoxins (B1, B2, G1 and G2) in baby food.

## 1. Introduction

Mycotoxins are secondary metabolites produced by a wide variety of fungal species and constitute a significant hazard to the food chain [1]. According to the Food and Agricultural Organization (FAO), at least 25% of the world’s food crops are contaminated with mycotoxins [2]. Mycotoxin production in agricultural commodities is greatly influenced by environmental factors, such as temperature, water activity, agronomical techniques, insect damage, drought, and inadequate storage conditions [3]. Among mycotoxins, two of the most relevant fungal toxins of food concern are aflatoxins (AFs) and deoxynivalenol (DON).

Aflatoxins are produced primarily by the fungus *Aspergillus flavus* and the closely related species *A. parasiticus. A. flavus* produces aflatoxins B1 and B2, while *A. parasiticus* produces B1, B2, G1 and G2. Aflatoxins are commonly present in different commodities such as nuts, cereals, dried fruits and spices [4]. Aflatoxin M1 is a metabolite that can occur in milk following exposure of lactating animals to aflatoxin B1 present in feedstuffs. The aflatoxins (B1, B2, G1, G2, M1) are classified as carcinogenic to humans (Group 1) by the International Agency for Research on Cancer [5].

Deoxynivalenol (DON) belongs to the type B trichothecenes, and it shows a widespread occurrence in cereals such as wheat, maize, barley, oat, rice, rye, millet and sorghum, as well as in cereal products (bread, pasta, breakfast cereals and infant cereal). *Fusarium graminearum* and *Fusarium culmorum* are the main molds responsible for DON contamination, whose toxicity is related to immunosuppressive and digestive effects in animals and humans [6].

Both aflatoxins and deoxynivalenol can occur in a wide range of agricultural commodities. Deoxynivalenol is primarily produced at pre-harvest in the field, while the aflatoxins typically occur at post-harvest during storage. Due to their stability during industrial processing, they may also be present in derived products such as processed cereal-based foods and baby foods for infants and young children. Not in vain, in the last five years the Rapid Alert System for Food and Feed of the European Commission (RASFF) registered 44 notifications for aflatoxins in cereals and bakery products and two in baby food, while for DON there were 28 notifications in cereals and bakery products [7]. For the aflatoxins, the alerts affecting baby foods were notified by Switzerland (aflatoxin B1 at 0.18 µg/kg) and The Netherlands (aflatoxin B1 from 0.21 to 0.39 µg/kg).

During the first several months of life, by ages 4–6 months, infants require the gradual replacement of breast milk with infant cereal products, which are frequently the first solid meal used in infant feeding [8]. Initially, gluten free infant cereals made of rice and maize are provided to infants, and thereafter multi-cereal based products are gradually administered. Consequently, aflatoxins and deoxynivalenol exposure seems to be unavoidable, since multiple susceptible commodities are present in baby foods, such as mixed-grain cereals. Infants may be more susceptible to mycotoxins because their rapid growth rate that makes the consequences of protein synthesis inhibition (DON) or liver damage (aflatoxins) more important than in adults. In addition, infants have increased vulnerability to contaminants than adults due to their lower body weight, reduced ability to detoxify hazardous agents, and more restricted diet [9].

According to the risks associated with mycotoxins in infants, the European Union has established the strictest maximum levels for aflatoxin B1 (0.10 µg/kg) and deoxynivalenol (200 µg/kg) in infant and baby foods in Commission Regulation (EC) No 1881/2006 [10]. However, no maximum levels have been established for the sum of aflatoxins B1, B2, G1 and G2, despite aflatoxins are considered carcinogenic to humans [5] and the four major aflatoxins may co-occur in infant cereals. Most baby foods for infants and young children are multi-cereal, as many different grains are used as ingredients, so increasing the probability of multiple mycotoxins. Therefore, in order to protect vulnerable population groups, surveillance studies are needed to determine the extent of contamination with the four aflatoxins and DON in foods intended for infants and young children.

Unfortunately, the availability of mycotoxin data for baby foods is rather limited in the recent years in Europe. In the present decade, there have been few surveys on the occurrence of aflatoxins and DON in infant foods in Europe [11,12,13,14,15,16]. In Spain, Hernández and Navarro [12] analyzed 91 baby food samples for aflatoxins and found 66 positive (72.5%), of which 7 samples (7.7%) exceeded the maximum level of 0.10 µg/kg for aflatoxin B1. In turn, Cano-Sancho et al. [13] reported no positive samples for aflatoxins in a study of 72 composite samples of powdered infant formula. However, aflatoxins were detected in breakfast cereals, corn snacks and sweet corn. The Spanish Organization of Consumers and Users (OCU) analyzed 15 samples of infant cereals and four were positive for aflatoxins [17]. Therefore, the presence of aflatoxins is relatively frequent in infant cereals, although generally at low levels, so we cannot lower our guard at any time.

Consequently, the aims of the present study were: (1) to determine the occurrence and levels of the four major aflatoxins and deoxynivalenol in cereal-based baby foods available in the Spanish market, (2) to compare with the maximum levels established by the EU regulations, and (3) to estimate the daily intake in different stages of infancy.

## 2. Results and Discussion

### 2.1. Performance and Validation of HPLC Analytical Method

The linearity for each aflatoxin (B1, B2, G1 and G2) was between 0.02 and 0.15 ng/mL, while for deoxynivalenol (DON) ranged from 30 to 500 ng/mL. The linearity requirements were met as the linear regression analysis data for the calibration curves of all studied mycotoxins produced coefficients of determination (R^2^) above 99%. The limit of detection (LD) for each aflatoxin (B1, B2, G1 and G2) was 0.02 µg/kg, and the limit of quantification (LQ) amounted to 0.06 µg/kg for each aflatoxin as well. For deoxynivalenol, LD and LQ were 33 and 100 µg/kg, respectively.

The recovery percentages for aflatoxins in spiked samples ranged from 91 to 99%, and for DON were between 89 and 96%. All recovery values were within the requirements of Commission Regulation (EC) No 401/2016 [18] that states recovery values of 50–120% for AFs and 60–110% for DON. The precision of the method was deemed adequate in terms of RSDr (repeatability) and RSDR (reproducibility) values, which were equal or less than 7%, falling within the established guidelines (Table 1).

These results confirmed that the analytical methods fulfilled the performance criteria set out in Commission Regulation (EC) No 401/2006 in terms of recovery, repeatability and reproducibility for both aflatoxins and deoxynivalenol in baby food. Likewise, the method was considered compliant with other European guidance documents, as repeatability in terms of RSDr (%) was lower than 20% and the recovery fell into the range 70–120% [19].

The authors’ laboratory participated in a worldwide interlaboratory comparison organized by Romer Labs during October 2017 (Ref. CSSMY013–M17411A). Our laboratory received a corn test material that was analyzed by HPLC-FLD-PHRED. Our results were satisfactory for all aflatoxins in terms of z-scores: aflatoxin B1 (−1.3), aflatoxin B2 (−1.0), aflatoxin G1 (−1.1) and the sum of aflatoxins (−1.3). A z-score between +2 and −2 is considered satisfactory performance, between +2 and +3 or between −2 and −3 is considered questionable performance, and anything outside this range (>+3 or <−3) is considered unsatisfactory [20].

### 2.2. Occurrence of Aflatoxins and Deoxynivalenol in Commercial Samples of Baby Food

The validated methods were used to determine the natural occurrence of the four major aflatoxins (B1, B2, G1 and G2) and deoxynivalenol (DON) in the samples of cereal-based baby food. From the total of 60 analyzed samples, aflatoxin B1 was detected in 11 samples (18.3%), aflatoxin G1 in 6 (10%), while aflatoxins B2 and G2 were only detected in one sample (1.7%) (Table 2). The sum of aflatoxins occurred in 12 samples (20%) and these contaminants were present in 9 out of 13 commercial brands of baby food. There were 48 samples, in which aflatoxins were not detected with the applied method of extraction and HPLC analysis. The aflatoxins B1 and G1 co-occurred in five samples, whereas one sample based on rice and quinoa contained aflatoxins B2, G1 and G2 but not aflatoxin B1. The co-occurrence of B and G aflatoxins may be related to the presence of *A. parasiticus* in the raw materials. The food-related hosts of *A. parasiticus* are similar to those of *A. flavus*, except that *A. parasiticus* is very uncommon in maize [5]. However, the distribution of toxigenic fungi in agricultural commodities and foodstuffs is affected by agronomic and climatic conditions that explain the differences in mycotoxin contamination among countries, different cereals and crop years.

The concentrations of aflatoxins in positive samples ranged from 0.08 to 0.23 µg/kg. The maximum concentration of aflatoxin B1 (0.23 µg/kg) showed in a sample based on wheat flour with cacao. In total, there were six samples exceeding the maximum level for aflatoxin B1 in processed cereal-based foods and baby foods for infants and young children (set at 0.10 µg/kg) (Table 2). It is worth noting that six baby food samples contained aflatoxins G1, B2 and G2, which maximum levels in infant cereals are not yet established by current EU regulations. Additionally, the levels of these non-regulated aflatoxins were above 0.10 µg/kg in all cases. The IARC (International Agency for Research on Cancer) has determined that the toxicity of aflatoxins follows the order AFB1 > AFG1 > AFB2 > AFG2 [21] and the occurrence in our samples followed the same sequence. This should be taken into account to study the establishment of the strictest maximum level for the sum of aflatoxins B1, B2, G1 and G2 in processed cereal-based foods and baby foods for infants and young children.

Regarding deoxynivalenol, the positive samples were 12 out of 60 (20%) (Table 2) and this mycotoxin was present in 7 out of 13 brands of infant cereals. It is remarkable that aflatoxins and DON did not co-occur in any sample. This fact was not related to the greater susceptibility of maize to aflatoxins and wheat to DON, because both mycotoxins appeared indistinctly in the samples regardless of whether they were based on corn or wheat. The reason for the non-co-occurrence may lie in the origin of the raw materials, because of diverse agronomic, climatic and storage practices.

As shown in Table 2, DON concentrations varied between 36 to 245 µg/kg, with a mean value of 117 µg/kg. The highest value (245 µg/kg) was reported in a sample containing rice and oat as main ingredients. Only one sample surpassed the maximum level for DON established at 200 µg/kg. There were 48 samples, in which DON was not detected with the applied method of extraction and HPLC analysis.

It is well known that all cereal types are susceptible to aflatoxins and deoxynivalenol. Ten samples of gluten-free cereals for infants under six months were positive for at least one mycotoxin (4 for aflatoxins and 6 for DON). These included products containing the cereals rice (10 samples), maize (7), millet (1) or quinoa (1). Likewise, 14 samples of multi-cereals for infants above six months were positive for at least one mycotoxin (8 for aflatoxins and 6 for DON). Positive samples within this group contained the cereals wheat (11 samples), rice (10), oat (10), barley (9), rye (8), corn (7), millet (6), sorghum (3), buckwheat (2), and spelt (1). Regarding other ingredients, cocoa was present in four aflatoxin-positive multi-cereal samples, while honey appeared in two of them. Therefore, different ingredients may be involved in aflatoxin contamination of baby food.

For comparative purposes, the samples were classified as gluten-free (*n* = 27) or multi-cereals (*n* = 33) according to the cereals on which they are based. Gluten-free cereals are intended for babies from 4 to 6 months and primarily based on maize, rice and millet, while multi-cereals (for babies aged 7 to 12 months) also contain wheat, barley, oat, spelt and rye. The contamination pattern was not very different between both sample types (Table 2), so there were no statistically significant differences for the aflatoxins (*p* > 0.05) or deoxynivalenol (*p* > 0.05).

In respect of the production system, there were 23 samples containing organic cereals (as indicated in the label) and the rest (37 samples) were made from conventional cereals. The incidence of aflatoxins was lower in organic samples (only 2 positives) than in conventional ones (10 positives), while the incidence of DON was equally distributed between organic (6 positives) and conventional samples (6 positives) (Table 2). The concentration of aflatoxins was similar in organic and conventional samples, while DON was significantly higher in organic (49 µg/kg) than in conventional baby foods (29 µg/kg) (*p* < 0.05).

Finally, the samples of baby food were also classified in two groups according to the presence of whole-grain ingredients (24 samples) or refined ingredients (36 samples). There were no remarkable differences between both groups (Table 2), except for deoxynivalenol, which mean levels were somewhat higher in integral baby foods (49 µg/kg) than in refined ones (28 µg/kg), but this difference was not confirmed statistically (*p* > 0.05).

The incidence of aflatoxins in our baby food samples were 18.3% (B1), 10% (G1), and 1.7% (B2 and G2). In comparison with other studies on baby food worldwide, our aflatoxins incidence percentage was lower than 66, 87 and 50% reported in Spain in 2010 by Hernández and Navarro [12], in Turkey in 2007 by Baydar et al. [22] and in Canada in 2006 by Tam et al. [23], respectively. In contrast, other researchers found lower incidence values of 7.1% in Brazil in 2017 [24] and 7% in Portugal in 2010 [25], while aflatoxins were not detected by Juan et al. in Italy in 2014 [14], Zhang et al. in the USA in 2014 [26] and Beltrán et al. in Spain in 2011 [27].

In agreement with our results, the presence of the four aflatoxins has been previously reported in the USA in 2017 by Al-Taher et al. [8], who found incidences of 4.7% for B1, 21.9% for B2, 14.1% for G2 and 0% for G1 in a study on 64 samples of infant cereals. It is noteworthy that the incidence of aflatoxins B2 and G2 was higher than that of aflatoxin B1. Perhaps, this is another example to support the study of the establishment of maximum levels for said aflatoxins in baby foods. From a toxicological point of view, considering that the aflatoxins are carcinogens, it would be appropriate to limit the total aflatoxin content of baby food (sum of aflatoxins B1, B2, G1 and G2), now that more data on their presence have become available.

In our research, 6 out of 60 analyzed baby food samples exceeded the maximum level for aflatoxin B1. These results are in agreement with those of Hernández and Navarro [12] who found 7 out of 91 Spanish infant cereals that surpassed the maximum AFB1 content (0.12 to 3.11 µg/kg). However, our level of non-compliant samples for aflatoxin B1 was higher than that reported in infant cereal across Canada in which only 2 out of 177 samples exceeded 0.10 µg/kg [23].

All cereal products used as ingredients in baby food are susceptible to the mycotoxins under study. The contamination with aflatoxins was mostly associated with the presence of rice and corn. According to the reported literature, these cereal grains are frequently involved in aflatoxin contamination [28,29,30]. Additionally, cocoa and honey, which are less susceptible to aflatoxin contamination, may also be present in positive baby food samples [12]. Cocoa and its derivatives can be a source of aflatoxins in the diet, which is a concern since babies and children are regular eaters of products that contain chocolate [31]. Therefore, monitoring all these susceptible ingredients in commodities intended to infant population is an important issue. Our results highlight the need for routine analysis of ingredients and final products to maintain aflatoxin levels as low as reasonably achievable in infant cereals.

Deoxynivalenol was detected in 20% of analyzed baby food samples. The incidence of DON was comparable to that of 26% found in Spain in 2012 by Rubert et al. [32], but lower than the 76, 65, 57 and 44% reported between 2014–2017 by Juan et al. [14], Sartori et al. [24], Zhang et al. [26] and Pereira et al. [33], respectively. However, the concentrations of DON were in line with other studies carried out in Italy in 2014 [14] and Portugal in 2015 [33].

In 2017, EFSA published a scientific opinion about risks to human and animal health related to the presence of DON in food and feed. The EFSA opinion pointed out that the occurrence data for DON in ‘food for infants and young children’ was particularly scarce. In this respect, it was noted that some articles provided only limited information, which sometimes made their interpretation difficult [34]. In our study, 12 out of 60 samples were positive for DON. These included baby foods containing rice (9 samples), corn (8), wheat (4), oat (3), millet (2), barley (2), rye (2), spelt (1) and buckwheat (1). For comparison, Juan et al. [14] detected DON in 19 out of 25 Italian samples of infant formula, and reported wheat, rice and maize as the main ingredients in positive samples.

There is a widespread belief that organically grown agricultural commodities may have higher incidence and mycotoxin levels than conventionally grown crops [35]. However, comparative studies in cereal crops are variable and controversial [36]. Some studies reported that products consisting of organic cereals may have higher incidence of deoxynivalenol and aflatoxin than conventional ones [12,37], but other works suggested that conventionally and organically cereal-based products showed similar occurrence of mycotoxins [38,39]. Relatively few studies have evaluated the differences in mycotoxin levels between conventional and organic baby foods. In our study, the concentration of aflatoxins was similar in organic and conventional samples, while DON was significantly higher in organic than in conventional baby foods. In the US, the study by Cappozzo et al. [40] showed similar incidences of ochratoxin A contamination in conventional and organic infant cereals.

### 2.3. Estimation of Daily Intakes for Aflatoxins and Deoxynivalenol

In our study, the daily dietary intakes for aflatoxins and deoxynivalenol were estimated for four age groups: 4 months, 5 months, 6 months and 7–12 months. The estimated daily intakes (EDIs) were calculated as indicated in the following equation:

(1) EDI=(K(g/day)×Cm(µg/kg))/bw(kg)

where EDI is the estimated daily intake for aflatoxins (ng/kg bw/day) and deoxynivalenol (µg/kg bw/day); K is the cereal intake (g/day) for each age group; Cm is the mean concentration of mycotoxins (µg/kg); bw (kg) is the body weight at different ages of infancy.

The cereal intakes were 48 g/day for babies aged 4 months, 60 g/day for 5 months of age, 72 g/day for 6 months of age, and 84 g/day for infants aged 7 to 12 months [12]. The mean concentrations of mycotoxins were taken from Table 2, using the mean value of gluten-free cereals for babies aged 4 to 6 months, and that of multi-cereals for infants from 7 to 12 months of age. The mean body weight was 6.5 kg (4 months), 7.25 kg (5 months), 7.75 kg (6 months) and 9 kg (7–12 months), as indicated by Hernandez and Navarro [12].

**Figure 1:**
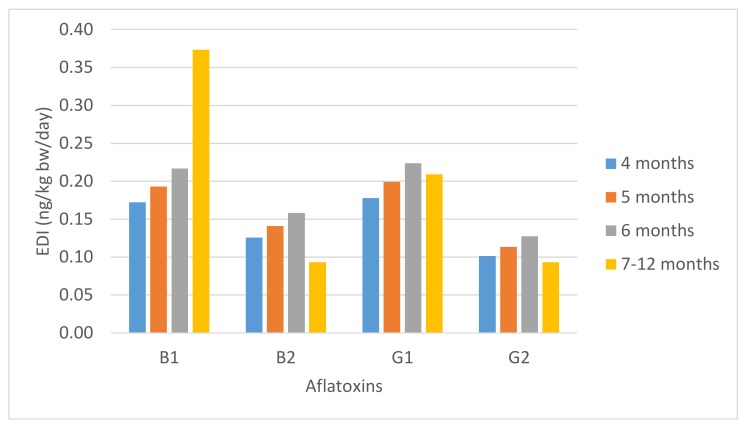
Estimated daily intake of aflatoxins (ng/kg bw/day) from cereal-based baby food in different stages of infancy.

Figure 1 shows the estimated daily intake (EDI) for each aflatoxin from cereal-based baby food in different stages of infancy. The EDIs for AFB1 ranged from 0.17 to 0.37 ng/kg bw/day, for AFG1 between 0.18 and 0.21 ng/kg bw/day, and for AFB2 and AFG2 were 0.09–0.13 ng/kg bw/day. Our results were in accordance with the study of Bakker et al. [41] in The Netherlands, which estimated an EDI of 0.42 ng AFB1/kg bw/day for children of 2–6 years old.

As aflatoxins are carcinogenic to humans (Group 1 of IARC), it is assumed that there is no threshold of exposure and no tolerable daily intake (TDI) can be established. In these cases, “zero risk” could be achieved only by eliminating all possible human exposure. However, this may not be possible with naturally occurring carcinogens, such as the aflatoxins that may be widespread in many foodstuffs. Therefore, it is desirable to reduce exposure to carcinogens to as low as reasonably achievable (ALARA principle), while recognizing that “zero” exposure is impossible.

The risk exposure to aflatoxin B1 was expressed as a margin of exposure (MOE) that is calculated as the ratio between the toxicity effect level (benchmark dose, BMD) and the estimated exposure dose (EDI). The EFSA has suggested a benchmark dose lower confidence limit 10% (BMDL10) for aflatoxin B1 of 170 ng/kg bw/day, based on carcinogenicity data in rats [42]. Then, calculated MOE values ranged from 459 to 1000, which may represent a health risk, as in general a MOE ≥ 10,000 is considered to be protective [42]. Hernández and Navarro [12], according to literature review, affirmed that the daily exposure to aflatoxins of European infants (<1 year age) has been reduced nearly 10-fold from 2.4–4.5 ng/kg bw/day in the 1990s to 0.32 ng/kg bw/day in recent times. This can be due, in part, to the strict maximum levels established by Commission Regulation (EC) No 1881/2006 setting maximum levels for certain contaminants in foodstuffs. This regulation says that it is essential, in order to protect public health, to keep contaminants at levels which are toxicologically acceptable. Maximum levels should be set at a strict level that is reasonably achievable by following good agricultural, storage and manufacturing practices and taking into account the risk related to the consumption of the food.

**Figure 2:**
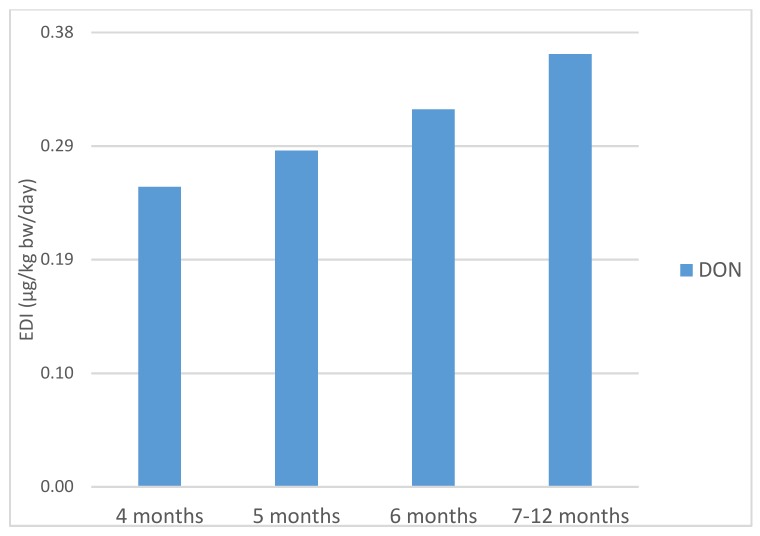
Estimated daily intake of deoxynivalenol (μg/kg bw/day) from cereal-based baby food in different stages of infancy.

IARC has categorized deoxynivalenol as Group 3, not classifiable as to its carcinogenicity to humans [21]. For this mycotoxin, a tolerable daily intake (TDI) has been established at 1 µg DON/kg bw/day by EFSA [34]. The estimated daily intakes for DON through the consumption of the infant cereals at 4, 5, 6 and 7–12 months were 0.25, 0.28, 0.32 and 0.36 µg/kg bw/day, respectively (Figure 2). These intakes were respectively 25.1, 28.1, 31.6 and 36.2% of the TDI (Tolerable Daily Intake) established by EFSA. Our results were higher than 0.08 µg DON/kg bw/day reported for cereal-based baby foods for infants in Spain [39].

## 3. Conclusions

The results of the present study, carried out in Spain, provide additional information related to the occurrence and exposure assessment of aflatoxins and deoxynivalenol in cereal-based baby foods. Forty percent of infant cereal samples were contaminated with at least one of the analyzed mycotoxins. In total there were six samples exceeding the EU maximum level for aflatoxin B1, while only one sample surpassed the EU maximum level for DON. The four major aflatoxins (B1, B2, G1 and G2) co-occurred in several samples. Therefore, infants and young children, a vulnerable group, are exposed to the different carcinogenic aflatoxins, what is explained by their restricted diet based on different cereal types. The margin of exposure (MOE) to aflatoxin B1 may represent a health risk, as it was below the safe value of 10,000 that can be considered protective. In parallel, the estimated intake of deoxynivalenol amounted to nearly 36% of the TDI established at 1 µg/kg bw/day.

It is essential to keep these contaminants at levels toxicologically acceptable. Our results highlight the need for manufacturers to apply all conceivable measures through their HACCP procedure to prevent and reduce the contamination of raw materials used for the manufacturing of foods for infants and young children. Likewise, coordinated surveillance programs should ensure the compliance with the maximum levels set for mycotoxin in processed cereal-based foods and baby foods for infants and young children. The establishment of the lowest maximum level for the sum of aflatoxins B1, B2, G1 and G2, should be also considered for the health protection of this vulnerable group.

## 4. Materials and Methods

### 4.1. Samples

Sixty samples of infant cereals were randomly collected from different supermarkets, pharmacies and organic food retailers in Cantabria and Aragón (Spain) during 2016 and 2017. The samples were from 13 brands (9 conventional and 4 organic manufacturers) that can represent the majority of market share in Spain of these products. The samples were classified into two groups according to the age of the children they were intended for: (1) gluten-free samples for babies from 4 to 6 months of age (*n* = 27), and (2) multi-cereals for infants aged 7 to 12 months (*n* = 33). According to the ingredients declared on the label, gluten-free infant cereals were basically composed of one or two cereals among maize, rice and millet, and occasionally quinoa, cassava and buckwheat. Baby food based on multi-cereals contained a wide variety of grains such as wheat, barley, oat, spelt, rye, sorghum, maize and rice. Some of these samples also contained other minor ingredients such as cocoa and honey. From the total 60 samples, 37 were sold as conventional and 23 as organic, while 24 contained whole grain cereals as ingredients and 36 samples were made with refined cereals.

### 4.2. Chemical and Reagents

Sodium chloride (NaCl) was obtained from Panreac (Barcelona, Spain) and phosphate-buffered solution (PBS) was provided by Sigma-Aldrich (St Louis, MO, USA). The immunoaffinity columns (IAC) AflaTest WB SR and DonTest were purchased from VICAM (Watertown, MA, USA). Nitrogen (N_2_) C55 for solvent evaporation was obtained from Carburos Metálicos (Barcelona, Spain). Deionized water was obtained from a Millipore milli-Q water purification system (Mildford, MA, USA). HPLC grade acetonitrile (ACN) and methanol (MeOH) were obtained from Scharlau (Scharlab, Barcelona, Spain). Chromatographic solvents and water were degassed for 30 minutes using an ultrasonic bath Branson 3510 supplied by Selecta (Barcelona, Spain).

The stock solution of aflatoxins was purchased from Sigma-Aldrich (St Louis, MO, USA), and consisted of a mix with 1 µg AFB1, 0.3 µg AFB2, 1 µg AFG1 and 0.3 µg AFG2 in methanol. Deoxynivalenol stock solution at 200 µg/mL in ethyl acetate/methanol (95:5) was supplied from the same manufacturer. An intermediate solution for aflatoxins was made by 100-fold dilution of the original mix, then working standard solutions were prepared at 0.02 to 0.15 ng/mL for AFB1, G1, B2 and G2 in mobile phase consisting of water/acetonitrile/methanol (50:10:40). DON intermediate solution was made diluting stock solution 100-fold with methanol, and working standards were made at 30 to 500 ng DON/mL in mobile phase consisting of water/acetonitrile/methanol (95:5:5). Stock and intermediate standard solutions were stored at −20 °C, whereas working calibration solutions were stored at 4 °C and renewed every week.

As a safety note, all used laboratory glassware were treated with an aqueous solution of sodium hypochlorite (5%) before discarding to minimize health risks due to mycotoxin contamination [43].

### 4.3. HPLC Equipment and Chromatographic Conditions

The system consisted of an Agilent 1100 Series HPLC coupled to diode-array (DAD) and fluorescence (FLD) detectors (Agilent Technologies, Barcelona, Spain). Separation was carried out on a LC column Ace 5 C18, 250 × 4.6 mm, 5 µm particle size (Análisis Vínicos, Ciudad-Real, Spain) at 50 and 25 °C for aflatoxins and deoxynivalenol, respectively. A manual injector system, equipped with a 100-μL injector loop and a 250-μL syringe, was used. The isocratic mobile phases mentioned above were pumped at a flow rate of 0.7 mL/min for aflatoxins and 1.0 mL/min for DON. The FLD for aflatoxins was set at 365 nm (excitation) and 435 nm (emission), while the DAD for deoxynivalenol was set at 220 nm. The LC system was connected to a photochemical reactor for enhanced detection (PHRED) (LCTech UVE, Dorfen, Germany) set at 254 nm for post-column derivatization of aflatoxins.

### 4.4. Mycotoxin Analysis

Preliminary experiments were done to determine the applicability of standard methods for the determination of mycotoxins in cereal-based baby food. For aflatoxins, the method EN: 15851:2010 was used, whereas deoxynivalenol was determined by the method EN: 15891:2011, both adopted by the International Standard Organization [44,45]. The analytical methods were validated in-house in terms of linearity, sensitivity, accuracy and precision.

In short, method EN: 15851:2010 starts by mixing 5 g of sample with 0.5 g of sodium chloride into 50 mL centrifuge tube. For extraction, 20 mL of methanol/water (80:20) were added and vortexed (TK3S, Techno Kartell, Noviglio, Italy) for 3 minutes. The extract was filtered by Whatman paper number 1 (Symta, Madrid, Spain) and 4 mL of the filtrate was mixed with 16 mL of PBS solution and subjected to IAC clean-up with AflaTest WB SR (Vicam) according to manufacturer’s instructions. The collected methanolic extract was dried under a stream of N2 at 50 °C in a sample concentrator (Stuart instruments, Cambridge, UK), dissolved in 250 µL mobile phase and filtered with 0.45 µm filter (Análisis Vínicos, Ciudad-Real, Spain) immediately before injection into the LC-FLD-PHRED system.

The method EN: 15891:2011 for deoxynivalenol starts with five grams of sample vortexed with 20 mL of methanol for 3 minutes and filtered through Whatman filter paper number 4 (Symta, Madrid, Spain). The IAC clean-up with DonTest (Vicam) was carried out as per the instructions of the manufacturer. Two mL of the collected methanolic extract were evaporated under a stream of N2 at 50 °C and the dried residue was reconstituted with 250 µL of mobile phase, filtered through a 0.45 µm filter and injected into the LC-DAD system.

### 4.5. Method Validation

The analytical methods were validated in-house according to Commission Regulation (EC) No 401/2016 in terms of linearity, sensitivity (limits of detection and quantification), precision (repeatability and reproducibility) and percentage of recovery.

The linearity of the methods was assessed through the response of standard solutions of certified concentration for each of the mycotoxins tested, using three injections for each calibration level. The limits of detection (LD) and quantification (LQ) were determined for a signal/noise ratio of 3 and 10, respectively. The blank sample used for the evaluation of LD and LQ was a baby food not contaminated with aflatoxins and deoxynivalenol. This blank sample was spiked at different mycotoxin levels for recovery assays: 0.10, 0.20 and 0.40 µg/kg for total aflatoxins (sum of B1, B2, G1 and G2) and 100, 200 and 400 µg/kg for deoxynivalenol. The precision was evaluated in terms of relative standard deviation (RSD%) from independent replicate analysis both intra-day and inter-day.

In October 2017, the authors’ laboratory participated in a check sample survey (Ref. CSSMY013 – M17411A), organized by Romer Labs Holding GmbH in Austria. The participants (396 laboratories all over the world) received a sample of milled corn naturally contaminated with aflatoxins B1, B2 and G1.

### 4.6. Data Analysis

The identification and quantification of mycotoxins in the samples was performed using the software package OpenLAB CDS 2013 (Agilent). A sample was positive for a tested mycotoxin when the level was above the limit of detection (LD). Samples below the LD were assigned a value of one half the LD for the interpretation of the results and exposure assessment purposes [46]. Exceeding samples were those which mycotoxin concentration was above the maximum levels set out in Commission Regulation (EC) No 1881/2006. The descriptive analysis of mean, standard deviation (SD) and relative standard deviation (RSD%) was performed with Statistical Package SPSS v21 (IBM Corporation, Armonk, NY, USA). Statistical analysis was done using one-way analysis of variance (ANOVA) with the Least Significant Difference (LSD) post hoc test, with *p* < 0.05 to determine significant differences in the levels of mycotoxins between groups.

## Figures and Tables

**Table 1:** Recovery and precision of the analytical methods for total aflatoxins (the sum of B1, B2, G1 and G2) and deoxynivalenol in baby food (3 replicates at each spiking level).

Mycotoxin	Percentage of Recovery	Repeatability RSD_r_ %	Reproducibility RSD_R_ %
Aflatoxins			
0.10 µg/kg	91	5	6
0.20 µg/kg	97	3	6
0.40 µg/kg	99	2	5
Deoxynivalenol			
100 µg/kg	89	4	5
200 µg/kg	96	4	7
400 µg/kg	95	4	6

**Table 2:** Occurrence of aflatoxins and deoxynivalenol in different types of baby food. Concentration values as mean ± standard deviation (SD) expressed in µg/kg.

Baby Food	Descriptive	AFB1	AFB2	AFG1	AFG2	DON
Total (*n* = 60)	Positive	11	1	6	1	12
	Mean ± SD	0.03 ± 0.05	0.01 ± 0.02	0.02 ± 0.04	0.01 ± 0.01	37 ± 50
	Range	<LD ^1^—0.23	<LD—0.20	<LD—0.16	<LD—0.11	<LD—245
	Exceeding ^2^	6	no ML ^3^	no ML	no ML	1
Gluten-free 4–6 months (*n* = 27)	Positive	3	1	3	1	6
	Mean ± SD	0.02 ± 0.04	0.02 ± 0.04	0.02 ± 0.04	0.01 ± 0.02	34 ± 42
	Range	<LD—0.20	<LD—0.20	<LD—0.15	<LD—0.11	<LD—194
	Exceeding	1	no ML	no ML	no ML	0
Multi-cereals 7–12 months (*n* = 33)	Positive	8	0	3	0	6
	Mean ± SD	0.04 ± 0.06	<LD	0.02 ± 0.04	<LD	39 ± 56
	Range	<LD—0.23	-	<LD—0.16	-	<LD—245
	Exceeding	5	no ML	no ML	no ML	1
Conventional cereals (*n* = 37)	Positive	10	0	5	0	6
	Mean ± SD	0.04 ± 0.06	<LD	0.03 ± 0.05	<LD	29 ± 32
	Range	<LD—0.23	-	<LD—0.16	-	<LD—148
	Exceeding	5	no ML	no ML	no ML	0
Organic cereals (*n* = 23)	Positives	1	1	1	1	6
	Mean ± SD	0.02 ± 0.04	0.02 ± 0.04	0.01 ± 0.02	0.01 ± 0.02	49 ± 69
	Range	<LD—0.18	<LD—0.20	<LD—0.12	<LD—0.11	<LD—245
	Exceeding	1	no ML	no ML	no ML	1
Whole-grain ingredients (*n* = 24)	Positive	5	0	2	0	6
	Mean ± SD	0.04 ± 0.06	<LD	0.02 ± 0.04	<LD	49 ± 68
	Range	<LD—0.20	-	<LD—0.16	-	<LD—245
	Exceeding	3	no ML	no ML	no ML	1
Refined ingredients (*n* = 36)	Positive	6	1	4	1	6
	Mean ± SD	0.03 ± 0.05	0.02 ± 0.03	0.02 ± 0.04	0.01 ± 0.02	28 ± 32
	Range	<LD—0.23	<LD—0.20	<LD—0.15	<LD—0.11	<LD—148
	Exceeding	3	no ML	no ML	no ML	0

^1^ Limit of detection (0.02 µg/kg for each aflatoxin and 33 µg/kg for DON); ^2^ Exceeding the maximum levels (ML) set out in Regulation (EC) No 1881/2006 (AFB1 and DON); ^3^ There are no maximum levels established by Regulation (EC) No 1881/2006.
